# Stable Vascular Connections and Remodeling Require Full Expression of VE-Cadherin in Zebrafish Embryos

**DOI:** 10.1371/journal.pone.0005772

**Published:** 2009-06-03

**Authors:** Mercedes Montero-Balaguer, Kendra Swirsding, Fabrizio Orsenigo, Franco Cotelli, Marina Mione, Elisabetta Dejana

**Affiliations:** 1 FIRC Institute of Molecular Oncology, Milan, Italy; 2 Department of Biology, School of Sciences, University of Milan, Milan, Italy; 3 Department of Biomolecular Sciences and Biotechnologies, School of Sciences, University of Milan, Milan, Italy; Katholieke Universiteit Leuven, Belgium

## Abstract

**Background:**

VE-cadherin is an endothelial specific, transmembrane protein, that clusters at adherens junctions where it promotes homotypic cell-cell adhesion. *VE-cadherin* null mutation in the mouse results in early fetal lethality due to altered vascular development. However, the mechanism of action of VE-cadherin is complex and, in the mouse embryo, it is difficult to define the specific steps of vascular development in which this protein is involved.

**Methodology and Principal Findings:**

In order to study the role VE-cadherin in the development of the vascular system in a more suitable model, we knocked down the expression of the coding gene in zebrafish. The novel findings reported here are: 1) partial reduction of *VE-cadherin* expression using low doses of morpholinos causes vascular fragility, head hemorrhages and increase in permeability; this has not been described before and suggests that the total amount of the protein expressed is an important determinant of vascular stability; 2) concentrations of morpholinos which abrogate *VE-cadherin* expression prevent vessels to establish successful reciprocal contacts and, as a consequence, vascular sprouting activity is not inhibited. This likely explains the observed vascular hyper-sprouting and the presence of several small, collapsing vessels; 3) the common cardinal vein lacks a correct connection with the endocardium leaving the heart separated from the rest of the circulatory system. The lack of closure of the circulatory loop has never been described before and may explain some downstream defects of the phenotype such as the lack of a correct vascular remodeling.

**Conclusions and Significance:**

Our observations identify several steps of vascular development in which VE-cadherin plays an essential role. While it does not appear to regulate vascular patterning it is implicated in vascular connection and inhibition of sprouting activity. These processes require stable cell-cell junctions which are defective in absence of VE-cadherin. Notably, also partial modifications in *VE-cadherin* expression prevent the formation of a stable vasculature. This suggests that partial internalization or change of function of this protein may strongly affect vascular stability and organization.

## Introduction

Homotypic endothelial cell-cell adhesion is particularly important for the correct formation, networking and remodeling of vessels. Endothelial cells are maintained in contact one another by a complex network of transmembrane adhesion proteins anchored to the actin cytoskeleton. Growing evidence indicates that endothelial cell-cell adhesion is accompanied by intracellular signaling which promotes the resting state of the cells and contributes to their homeostasis [1]–. It is well known that cultured sparse and confluent cells present a different functional phenotype. Gene expression profiles of sparse and confluent cells also show that several genes are regulated by cell-cell contacts, many of which are implicated in cell growth, apoptosis, matrix and cytoskeletal remodeling [4]–[6].

Adherens and tight junctions (AJ and TJ respectively) are the major adhesive junctions in endothelial cells. They are both formed by transmembrane adhesive proteins that are linked inside the cells to a complex network of cytoskeletal and signaling partners [1], [7]–[8]. Adhesion at AJs is induced by members of the cadherin family. VE- and N-cadherin are the major cadherins present in endothelial cells. However, only VE-cadherin is found in the majority of inter-endothelial AJs both *in vitro* and *in vivo*.

Inactivation of *VE-cadherin* gene in mice leads to major defects in vascular development causing embryo lethality around 9.5–10.5 E [9], [10]. While the first primitive vascular plexus is formed even in the absence of VE-cadherin, the vessels are unable to further progress and the vasculature undergoes regression. The mechanism of action of VE-cadherin in vascular development is quite complex. Several reports indicate that, besides its adhesive properties, this protein can transfer intracellular signals which promote vascular stability and remodeling. Most of these activities, however, have been studied *in vitro* in cultured cells and, due to the early lethality of *VE-cadherin* null mouse embryos, it is difficult to understand how and at which step of vascular development this protein acts.

We therefore decided to use the zebrafish embryo which, due to its transparency and external development, allows a more detailed study of vascular defects [11]–[13]. Furthermore, only one *VE-cadherin* orthologue has been identified in the fish that maintains endothelial specific expression [14]. Our studies show that reduction of *VE-cadherin* expression does not change vascular patterning but leads to a concentration-dependent alteration in vascular integrity and lumen formation. A novel finding derived from the analysis of this phenotype is that the sprouting vessels lack the capacity to establish successful reciprocal contacts and subsequent fusion. When *VE-cadherin* expression is abrogated the heart remains disconnected from the peripheral vascular network and blood circulation is absent. When *VE-cadherin* expression is only partially reduced, the vessels are fragile and hemorrhagic, particularly in the head. These data support the notion that VE-cadherin plays a non redundant role in maintaining the integrity of endothelial cell-cell contacts and, as a consequence, in the establishment of correctly interconnected vascular structures. Notably, also partial modifications in *VE-cadherin* expression may prevent the formation of a stable vasculature suggesting that also small reduction in expression or function of this protein may severely affect vascular integrity.

## Materials and Methods

### Zebrafish lines and husbandry

Zebrafish (*Danio rerio*) were raised and maintained as described [15] in agreement with local and national sanitary regulations. Wild type AB line and transgenic lines Tg(*fli1*:EGFP)*^y1^* and Tg(*flk1*:EGFP)*^s844^* were used [16], [17]. The transgenic line Tg(*fli1*:EGFP)*^y1^* was used for general analysis of the vasculature. The possibility to breed this line in homozygosis allows stronger intensity of the fluorescent signal and better conditions for the acquisition of long time-lapse series. However, cranial vessels cannot be accurately imaged with the Tg(*fli1*:EGFP)*^y1^* line due to the strong expression of EGFP in branchial arches, therefore, in order to analyze the head vessels (cranial and aortic vessels), we used the Tg(*flk1*:EGFP)*^s844^* line.

Embryo staging was performed according to morphology as described [18].

### Morpholino injections and RT-PCR

**Figure 1:**
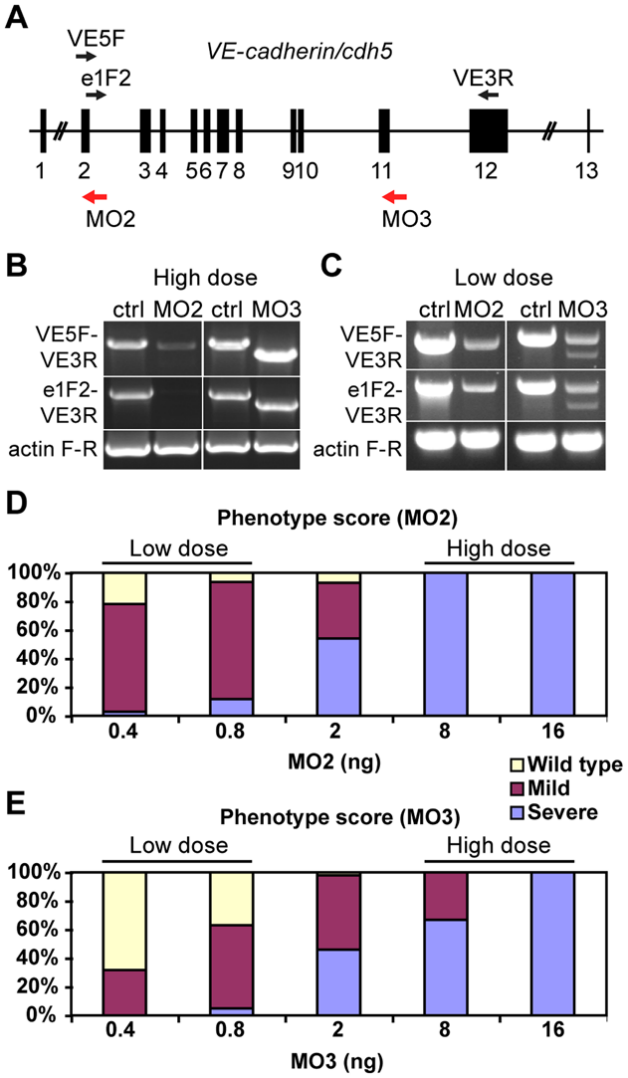
*VE-cadherin* morpholinos downregulate *VE-cadherin* expression in a dosage dependent manner. (A) Schematic representation of zebrafish *VE-cadherin*/*cdh5* gene with exons represented as black boxes. The positions of the morpholinos employed in this study are indicated with red arrows, and the primers used for the analysis of splice variants as black arrows (VE5F, e1F2 and VE3R). (B–C) Expression of *VE-cadherin* was analyzed by RT-PCR using two sets of primers. (B) Upon injection of high doses (16 ng) of *VE-cadherin* morpholinos, aberrant splicing of *VE-cadherin* was observed for both MO2 and MO3 when using primer pair VE5F-VE3R. Primer pair e1F2-VE3R allowed the detection of endogenous wild type *VE-cadherin* transcript in controls (Ctrl) and proved the absence of this transcript in MO2 injected embryos (left panel in B). This primer pair confirmed the altered splicing for MO3 injected embryos (right panel in B). Actin was used as an internal control. (C) Injection of low doses of morpholino (0.8 ng) caused partial knockdown of *VE-cadherin*. These embryos expressed both wild type *VE-cadherin* and aberrant spliced transcripts. (D–E) Histograms show the quantification of the effect of *VE-cadherin* morpholinos at 54 hpf. Both MO2 (D) and MO3 (E) cause increased severity of the phenotype increasing the doses injected. With low doses (0.8 ng) most of the embryos present circulation and hemorrhages (mild phenotype). Conversely, injection of high doses of either one morpholino (16 ng) cause a highly penetrant phenotype, with complete absence of blood circulation (severe phenotype). Intermediate amounts of morpholino lead to both mild and severe phenotype in different percentages. MO2 presented higher penetrance than MO3.

Two morpholino antisense oligonucleotides (Gene Tools) were designed to complement exon-intron boundaries of the zebrafish *VE-cadherin/cdh5* gene (diagram in figure 1A; Ensembl accession ENSDARG00000046128) in order to block splicing:

MO2 5′-TACAAGACCGTCTACCTTTCCAATC-3′;

MO3 5′- ATTTGAGATGAACCTACCCAGGATG-3′;

A 5 pb mismatch morpholino was used as control:

ctrlMO 5′-TAgAAcACgGTCTAgCTTTCgAATC-3′;

We used the morpholino against *silentheart* (*sih*)/*tnnt2* gene, that blocks translation of the thin-filament contractile protein cardiac troponin T2, as previously described [19]. We injected 4 ng of *sih*MO and confirmed the absence of the heart beat in *sih* morphants by direct observation of the embryos under the dissecting microscope at 26–30 hours postfertilization (hpf).

Morpholinos were reconstituted in nuclease-free water, and the volume of the injected drop was estimated with a micrometer scale. Embryos at 1- to 4-cell stage were injected with the indicated amounts of morpholino into the yolk immediately below the blastomeres. The efficacy of the morpholino was assessed by RT-PCR with Superscript One-step RT-PCR system (Invitrogen) and using as template total RNA from control or *VE-cadherin* morpholino injected embryos. RNA was extracted with Trizol from 48 hpf pooled embryos and treated with DNase. The primers used are:

VE5F 5′-GATGAAACAGTGTGCCAGAAG-3′;

VE3R 5′-AGGCAGAAGACAGGATGGAGA-3′;

e1F2 5′-TGTATGCGTATGAGGAAACAC-3′;

ActinF 5′-ACCTCATGAAGATCCTGACC-3′;

ActinR 5′-TGCTAATCCACATCTGCTGG-3′.

### Microangiographies

Tetramethylrhodamine dextran (molecular weight 2×10^6^, Molecular Probes) was dissolved in PBS at 20 mg/ml and microinjected into the sinus venosus of anesthetized zebrafish embryos at 52–54 hpf. For embryos that exhibited no circulation, microinjection was performed directly into the heart chambers. Images of the microangiograms were acquired within ten minutes upon injection.

### Microscopy and confocal imaging

For imaging zebrafish vessels, live embryos were dechorionated manually with forceps, anesthetized using 0.016% tricaine (Sigma) and mounted in 1.2% low melting agarose. Image acquisition was performed with 20× or 40× water immersion objective on a Leica TCS SP2 confocal microscope. Confocal stacks were processed for maximum intensity projections with Leica LCS software and movies were assembled using ImageJ (MacBiophotonics).

For video recordings of heart beat and circulation, embryos were anesthetized and mounted in 2% methylcellulose. Videos were acquired under an Olympus SZX12 stereomicroscope coupled with a JVC color video camera (Model TK-C148OBE) using Pinnacle Studio Plus 9.3 software. Movies were compressed with Quicktime.

### Transplantation experiments (mosaic analysis)

Cell transplantations were performed as previously described [15], [20]. Briefly, donor *Tg*(*fli1*:EGFP) embryos were microinjected at 1- to 4-cell stage with tetramethylrhodamine dextran (10,000 MW, fluoro-ruby, Molecular Probes) and 8 ng of MO2 for morphant into AB wild type transplants. At sphere stage donor and host embryos were dechorionated by a brief incubation with pronase (Sigma) and transferred to agarose wells. About 20 cells were transplanted at the margin of AB wild type host embryos and were incubated at 28.5°C for further development. Chimeric embryos were analyzed under a fluorescence stereoscope and images were aquired using a Nikon Eclipse 90i fluorescence microscope.

### TUNEL assay, immunocytochemistry and in situ hybridization

Whole mount TUNEL assay was performed with Apoptag Red In situ Apoptosis detection kit (Chemicon International). Immunocytochemistry was performed as previously described [21]. Mouse anti-human ZO-1 was clone ZO1-1A12 (Invitrogen). Whole-mount *in situ* hybridization was performed as previously described [22].

### Rescue experiments

A fusion construct containing murine *VE-cadherin* cDNA in frame with mCherry [23] at the C-terminus was subcloned into pTol*fli1*epDest vector under the endothelial *fli1* enhancer/promoter flanked by Tol2 transposable elements [24]. Coinjection of 0.4 ng of MO2, 20 pg of plasmid DNA and 20 pg of Tol2 transposase mRNA [25] was performed directly into the blastomere of 1-cell stage embryos. The phenotype of the injected embryos was scored at 54 hpf. To evaluate the extent of mosaicism we scored the average number of endothelial cells in a reference area (see figure S2B) using *tg*(*fli1*:nEGFP)*^y7^* transgenic embryos [20]. Then we scored the number of GFP-positive endothelial cells in 40 embryos of the same age co-injected with 20 pg pTol*fli*-EGFP (marking construct) and 20 pg pTol*fli-mVE-cadherin:mCherry* (rescuing construct).

## Results

### Partial reduction of VE-cadherin expression affects vascular integrity

To knock down VE-cadherin function in zebrafish we designed morpholinos (modified antisense oligonucleotides) targeting either exon 2-intron 2 boundary (MO2) or exon 11-intron 11 boundary of the gene (MO3) (Figure 1A). Expression of *VE-cadherin* upon morpholino injection was analyzed by RT-PCR using two sets of primers. We used different doses of morpholinos to be able to reduce *VE-cadherin* expression in a dose dependent manner. Upon injection of a high dose (16 ng) of both morpholinos, aberrant splicing of the *VE-cadherin* transcript was observed for MO2 and MO3 by RT-PCR with primer pair VE5F-VE3R (Figure 1B). Sequence analysis of the morphant transcripts confirmed that they encoded truncated versions of VE-cadherin. Primer pair e1F2-VE3R allowed the detection of endogenous wild type *VE-cadherin* transcript in controls and proved the absence of this transcript in MO2 injected embryos. In addition, this primer pair confirmed the altered splicing for MO3 injected embryos (Figure 1B).

**Figure 2:**
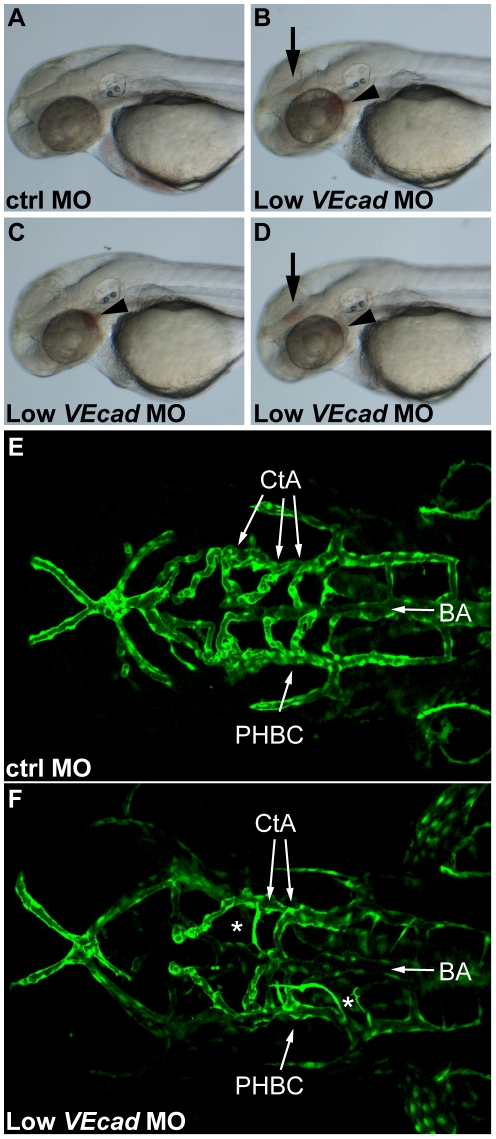
Partial *VE-cadherin* knockdown causes vascular instability and hemorrhages. (A–D) Bright field images of 52 hpf embryos. Lateral views, anterior to the left. Embryos injected with low doses (0.8 ng) of *VE-cadherin* morpholino (Low *VEcad*MO) develop single (C) or multiple hemorrhages (B and D) that were evident at 52 hpf and appeared in the brain (arrows) and/or aortic arches (arrowheads). No or very rare hemorrhages were observed in embryos injected with mismatch control morpholino (ctrlMO) at the same concentration (A). (E, F) Confocal images of Tg(*flk1*:EGFP) embryos at 54 hpf. Dorsal views, anterior to the left. In embryos injected with ctrlMO (E), central arteries (CtA) extend from the basilar artery (BA) towards forebrain and midbrain, and drain into the primordial hindbrain channel (PHBC). In contrast, in Low *VEcad*MO injected embryos (F) only few CtAs are formed, and those which are present fail to fuse to BA and lack lumen (asterisks).

At a low dose (0.8 ng) of both morpholinos *VE-cadherin* expression was partially knocked down but we could still detect endogenous wild type *VE-cadherin* transcript in addition to the alternatively spliced transcript (Figure 1C). At 30 hpf embryos injected with low doses of *VE-cadherin* morpholinos appeared morphologically normal (Movie S3) and essentially undistinguishable from ctrlMO injected embryo (Movie S1). They also presented regular heart beat, comparable to that of control embryos. However in 50% of these embryos, we could observe blood cells blocked in cranial vessels (see pulsating cells in Movie S3) impairing the correct completion of cranial circulatory loops. Due to this, the number of circulating blood cells in the trunk was reduced in these embryos (compare Movies S2 and S4). Subsequently, by 52–54 hpf more than 60% of embryos injected with low doses of either MO2 (0.4 ng, n = 127) or MO3 (0.8 ng, n = 102) presented a hemorrhagic phenotype (Figure 1D,E and figure 2B–D), not observed in control embryos (Figure 2A). The hemorrhages were particularly frequent intracranially and/or at the point of connection of aortic arches (AA) to the lateral dorsal aorta (arrows and arrowheads in figure 2B–D). These embryos were phenotypically classified as mild morphants. In 32% of the cases the same embryo presented more than one hemorrhagic foci (n = 33/102). The intracranial hemorrhages were localized randomly in forebrain, midbrain or hindbrain, and were equally present on the right or the left side. MO2 caused more penetrant alterations since at 0.8 ng, 80% of embryos showed hemorrhagic events (n = 297) while injection of the same dose of MO3 led to only 60% of morphants with hemorrhages (Figure 1D,E). By further increasing the doses, more severe alterations of vascular development were observed with both morpholinos. A higher dose of MO3 was needed for causing fully penetrant severe phenotype with complete absence of circulation (see below for full description). We therefore selected MO2 for further studies.

**Figure 3:**
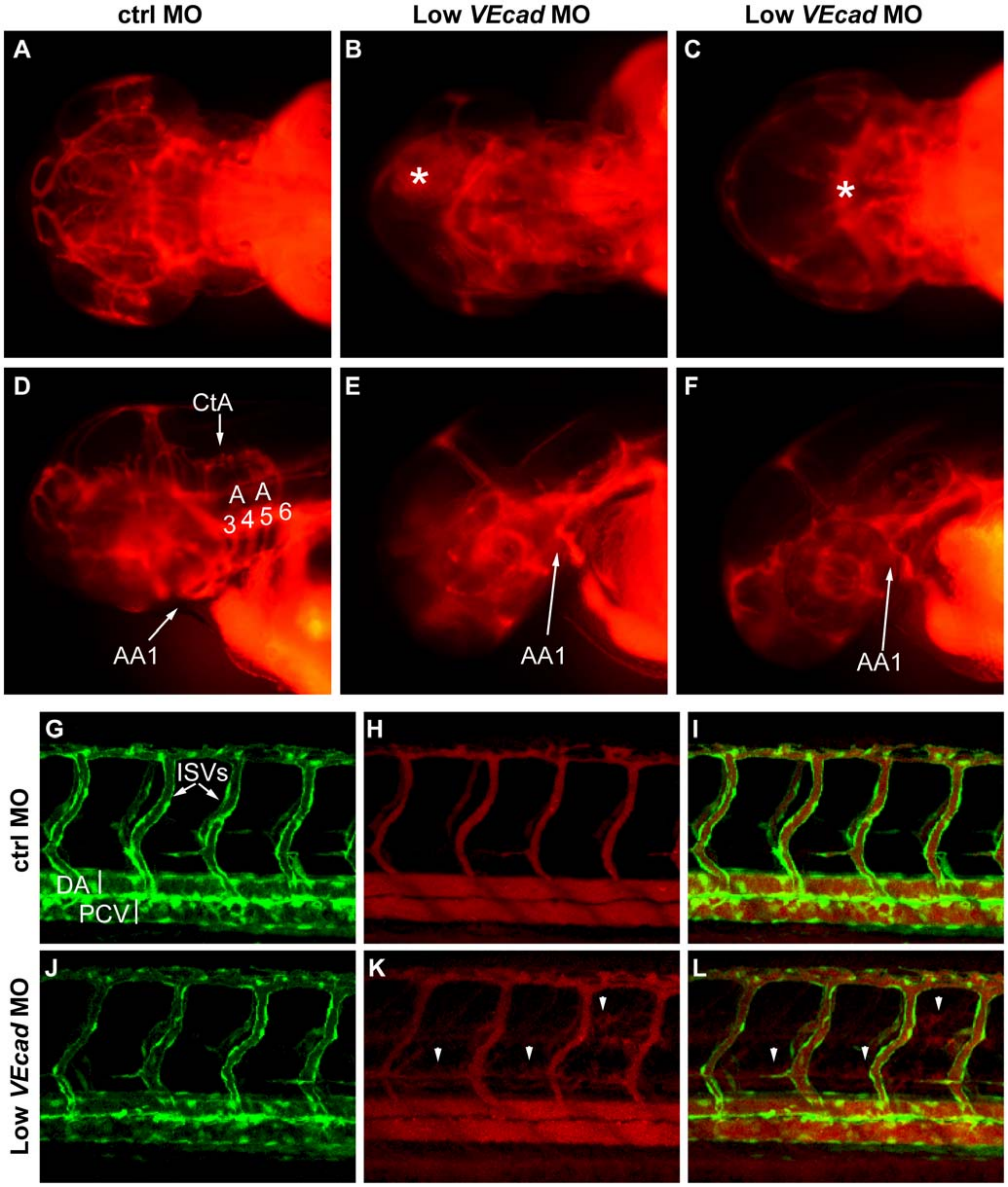
Partial *VE-cadherin* knockdown affects cranial and trunk vessel integrity. (A–F) Microangiographies performed at 52 hpf. (A–C) Dorsal views, (D–F) lateral views of the embryos above, anterior to the left. Control embryos (A, D) have a functional and complex cranial vascular network with lumenized aortic arches (AA) and central arteries (CtA). Microangiograms of embryos injected with low dose (0.8 ng) of *VE-cadherin* morpholino that developed hemorrhages (Low *VEcad*MO) show defects in head perfusion. B, E and C, F correspond to two different embryos with the same treatment. Extravasation of the injected dextran is observed at the site of the hemorrhages (asterisk in B and C). In addition, only the first aortic arch (AA1) is fully formed in the mild morphants but aortic arches 3 through 6 do not exhibit circulation (see panels E, F). (G–L) Microangiographies performed in Tg(*flk1*:EGFP) embryos at 54 hpf. Lateral views of trunk vessels are shown. (G, J) EGFP positive vessels, (H, K) microangiograms performed with rhodamine-dextran, and merge images (I, L). In control embryos (G–I) intersegmental vessels (ISVs), dorsal aorta (DA) and posterior cardinal vein (PCV) are fully lumenized and the injected dextran remains inside this primary vascular network. In some *VE-cadherin* mild morphants (Low *VEcad*MO) these vessels are apparently normal (J) but leakage is observed in the somites and regions surrounding the ISVs (arrowheads in panels K,L).

In order to analyze in more detail the organization of the vascular system, we used transgenic embryos expressing EGFP under the *flk1* promoter [17]. The dorsal view of the cranial vasculature showed that in 54 hpf embryos injected with ctrlMO, central arteries (CtAs) extended from the basilar artery (BA) towards the forebrain and midbrain and drain into the primordial hindbrain channel (PHBC) (Figure 2E). In contrast, embryos injected with 0.8 ng of *VE-cadherin* morpholino (*VEcad*MO) that developed hemorrhages presented marked alterations in vascular development (Figure 2F). In embryos that had hemorrhages in the midbrain, CtAs presented variable defects. On average two CtAs per embryo were missing and two presented very small diameter, lacked lumen and failed to fuse to the BA. These first observations suggested that the overall perfusion of the head was compromised. This was further confirmed by microangiographies performed at 52 hpf (Figure 3A–F). Embryos injected with ctrlMO have a functional cranial vascular network with lumenized aortic arches (AAs) and CtAs. Conversely, *VEcad*MO injected embryos lack perfusion in the brain and present extravasation of the injected dextran at the site of the hemorrhages (asterisks in figure 3B,C). In addition, only aortic arch 1 (AA1) is fully formed in the morphants, while AAs 3 through 6 do not exhibit circulation (Figure 3E,F).

Microangiographies were also performed to analyze the vessels of the trunk in Tg(*flk1*:EGFP) embryos at 54 hpf. In control embryos intersegmental vessels (ISVs), dorsal aorta (DA) and posterior cardinal vein (PCV) are fully lumenized, the injected dextran is confined to this primary vascular network and no leakage is observed (Figure 3G–I). In *VE-cadherin* mild morphants these vessels were apparently normal as detected by *flk1*:EGFP expression (Figure 3J). However, in 30% of these morphants we observed leakage of the fluorescent dye along the trunk vasculature in the somites and tissues adjacent to the ISVs (arrowheads in figure 3K,L).

### Strong or complete reduction of *VE-cadherin* expression impairs the development of a functional vascular network

**Figure 4:**
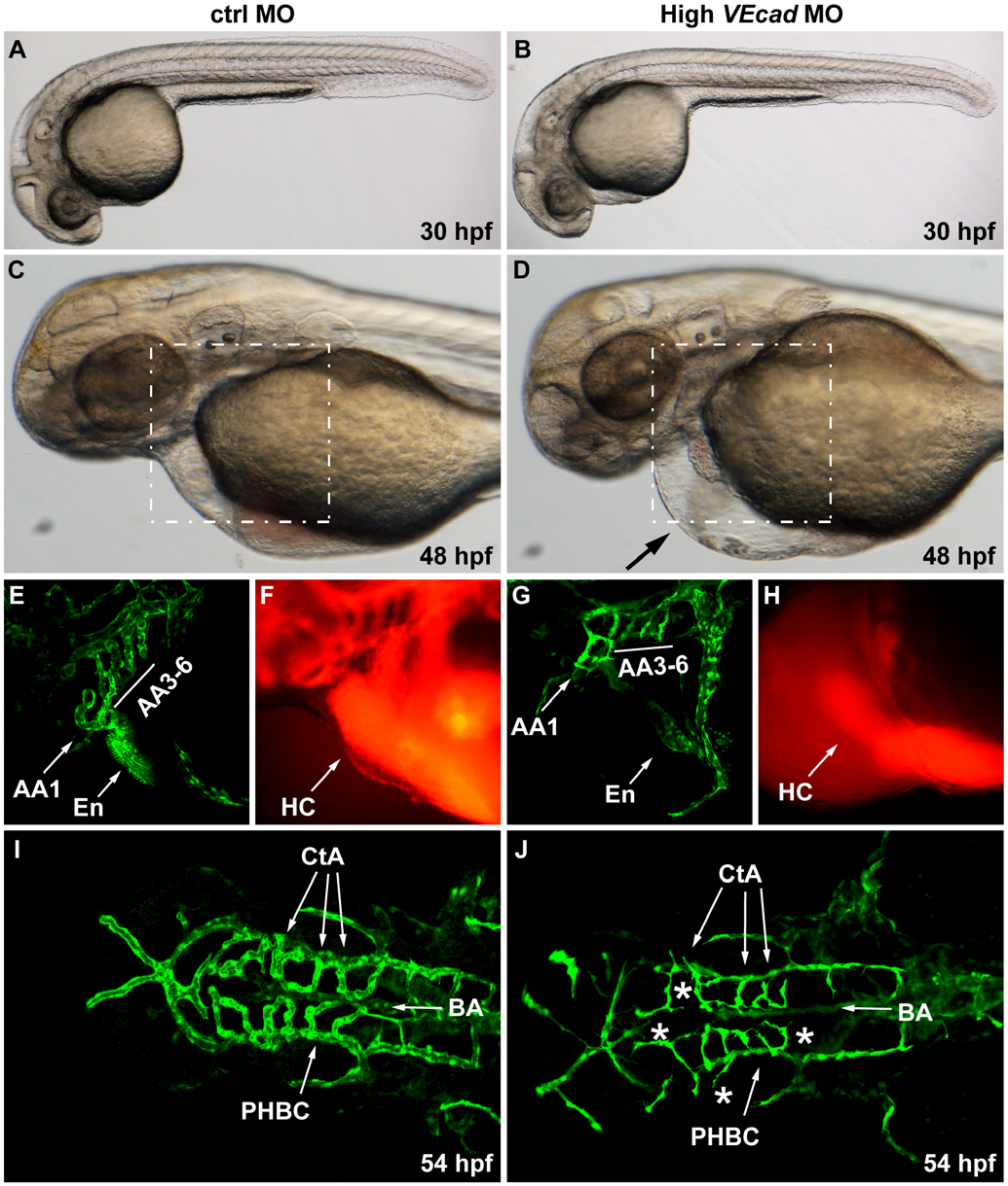
Complete VE-cadherin abrogation impairs the formation of a functional vascular network. (A–D) Bright field images of embryos injected with 16 ng of ctrlMO and *VE-cadherin* morpholino (High *VEcad*MO). *VE-cadherin* severe morphants present a normal morphological development at 30 hpf (B) but blood circulation is not established (see movies in supplementary material). At 48 hpf lack of circulation persists in this morphants with severe phenotype, and pericardial edema is observed (arrow in panel D). (E–H) Views of the endocardial and aortic arch region (highlighted box in C and D) at 52 hpf. Endocardium (En) and aortic arch vessels (AA) are visualized using a *flk1*:EGFP background (E, G) while microangiograms show interconnected lumenized vessels filled with rhodamine-dextan (F). *VE-cadherin* severe morphants form endocardium (En in panel G) that is not openly connected to the vasculature, neither to the aortic arch 1 (AA1) nor to the CCV, and retains the microinjected dextran in the heart chambers (HC). Rudiments of AAs without lumen are present in *VE-cadherin* morphants (G). (I, J) Dorsal views of Tg(*flk1*:EGFP) embryos at 54 hpf. In embryos injected with high dose of *VE-cadherin* morpholino (J) cranial vessels are apparently in place but central arteries (CtA) fail to form the connection to the basilar artery (BA), do not correctly lumenize and small sprouting vessels are present (asterisks).

Embryos injected with 16 ng of *VEcad*MO present a normal morphological development up to 30 hpf (Figure 4A,B). However blood circulation is not established (Movie S6) despite the regular heart beating (Movie S5), which indicates that the defect observed is not of cardiac origin. At 48 hpf lack of circulation persists in the morphants and is accompanied by pericardial edema (arrow in figure 4D), not observed in control embryos (Figure 4C).

Microangiographies on *flk1*:EGFP transgenic embryos injected with ctrlMO showed that the heart is interconnected with the AAs which, at 52 hpf, show a correct lumen (Figure 4E,F). In contrast, *flk1*:EGFP transgenic embryos injected with 16 ng of *VEcad*MO form the endocardial layer inside the heart (En in figure 4G), but connection with the AA1 and with the common cardinal vein (CCV, also called Duct of Cuvier) is missing and microinjected dextran is retained in the heart chambers (Figure 4H). Only rudiments of AAs without lumen are present in *VE-cadherin* morphants (Figure 4G).

Dorsal views of Tg(*flk1*:EGFP) embryos at 54 hpf show that *VE-cadherin* severe morphant embryos present cranial vessels which are in the correct place but show a very small diameter and fail to form a correct lumen (Figure 4J). Furthermore connections between the CtAs and the BA are defective and CtAs seem to interconnect one another instead. In addition small sprouting vessels along the length of few vessels could be observed (asterisks in figure 4J).

### VE-cadherin is required for the correct development of the common cardinal vein and for the closure of the circulatory loop

**Figure 5:**
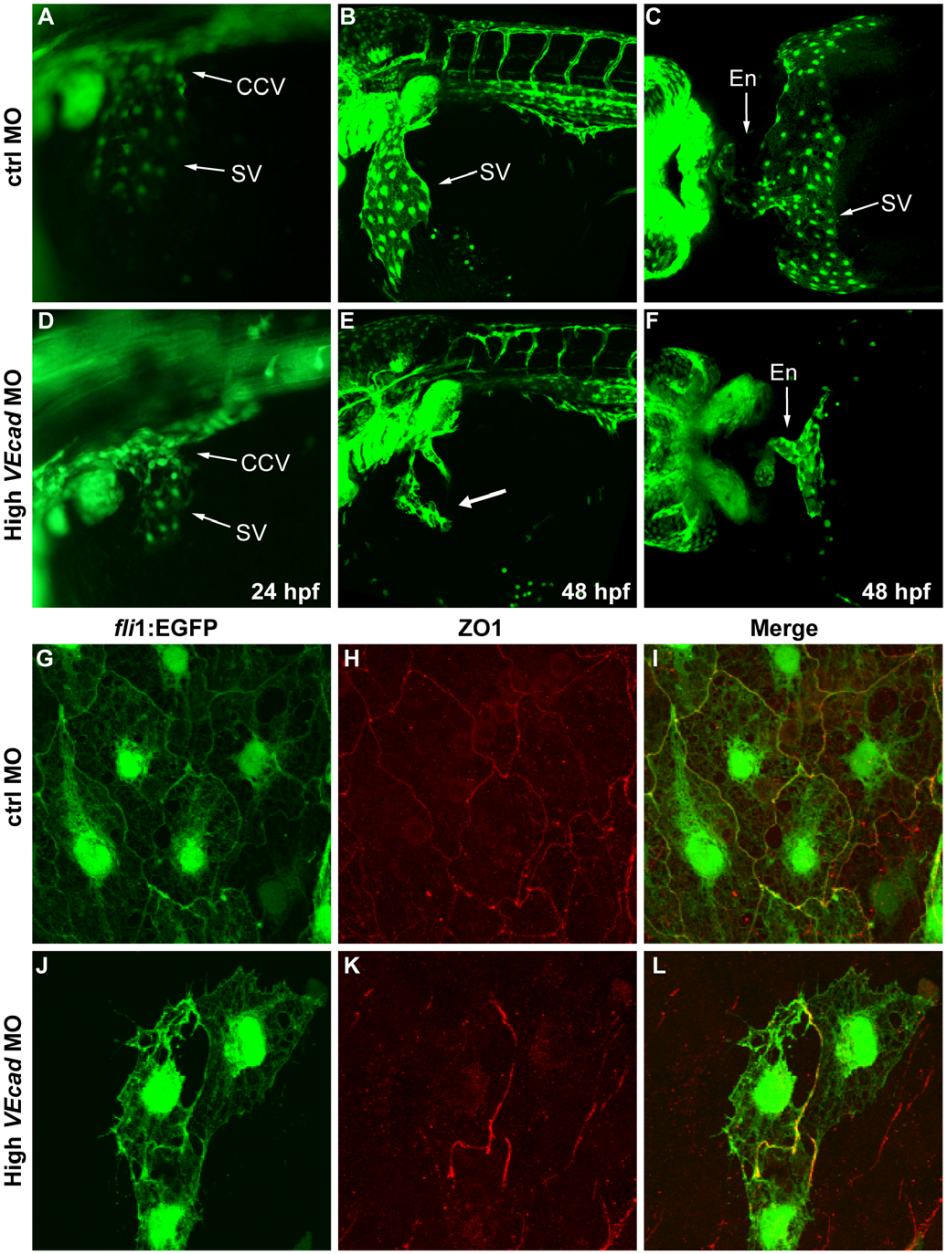
Depletion of VE-cadherin affects the formation of the sinus venosus. (A–F) Fluorescence images of *fli1*:EGFP embryos at the level of the common cardinal vein (CCV) and sinus venosus (SV). At 24 hpf a number of endothelial cells expand out of the CCV to form the SV both present in control embryos (A). In *VE-cadherin* severe morphant embryos (High *VEcad*MO) a rudiment of SV appears at 24 hpf (D). At 48 hpf the SV is fully formed in control embryos (B) while in high *VEcad*MO only few endothelial cells are present (arrow in panel E). Ventral views of fixed embryos allow the visualization of the endocardium (En) connected to a fully formed SV in control embryos (C). In contrast, high *VEcad*MO do have a tubular endocardium (En in panel F) but the SV is not formed. The absence of this structure impairs the connection of the CCV to the heart tube and the closure of the circulatory loop. (G–L) Immunofluorescence analysis by confocal microscopy of endothelial cells from the SV at 48 hpf. *Fli1*:EGFP positive endothelial cells visualized in G and J present intercellular junctions, marked by antibody staining against ZO1 (H, K). The few endothelial cells present in this SV region in high *VEcad*MO injected embryos show an aberrant morphology, but form some junctions at the sites of contact.

We then investigated why injection of high doses of *VEcad*MO severely impaired blood circulation. The CCV collects the blood from the trunk, head and the yolk sac and through the sinus venosus (SV) is connected to the heart. We therefore analyzed whether organization and remodeling of this vessel was affected by lack of *VE-cadherin* expression. Lateral views of *fli1*:EGFP control embryos at 24 hpf show that endothelial cells expand out of the CCV to form the SV which, within 48 hpf, is fully coated by endothelial cells and connected to the heart (Figure 5A–C). In *VE-cadherin* severe morphants while endothelial cells are still able to sprout from the CCV at 24 hpf, they are unable to further progress and form the SV and fail to establish a correct connection with the heart (Figure 5D–F). At 48 hpf severe morphants have only few endothelial cells where the SV should have been formed (arrow in figure 5E). The absence of this structure impairs the connection of the PCV to the heart tube and the closure of the first circulatory loop.

A closer analysis shows that endothelial cells in the SV of the morphants were greatly reduced in number and presented an altered morphology with strong cell retraction (Figure 5J). ZO-1 was still present in few areas of contact suggesting that other adhesive proteins at junctions such as claudins, JAMs or N-cadherin may still promote incomplete cell-cell adhesion (Figure 5J–L).

TUNEL staining of endothelial cells was limited to few cells (Figure S1) but was quite strong, as expected, in the unperfused tissues surrounding the ISVs and dorsal longitudinal anastomotic vessels (DLAV).

In order to confirm the specificity of the vascular phenotype observed upon injection of *VEcad*MO, we performed rescue experiments. In a first attempt we tried to rescue the severe phenotype using capped mRNA of zebrafish *VE-cadherin*. However, high doses of mRNA (100–400 pg) resulted in high mortality and abnormalities in the injected embryos. Low doses of *VE-cadherin* mRNA (20 pg) that did not cause abnormalities were not sufficient to rescue the phenotype when coinjected with *VEcad*MO (data not shown). We speculate that the overexpression of VE-cadherin during epiboly and gastrulation may have severe consequences in the adhesive properties of the cells at these stages because the morphogenetic movements required to form the different layers in the early embryo depend on a gradient of cell-cell adhesiveness [26]–[31]. This would explain the high mortality and morphological abnomalities that we observe upon ubiquitous overexpression of VE-cadherin. In order to achieve an endothelial specific rescue, we used a *fli1*:mVE-cadherin construct. The mCherry tag on the C-terminus of murine VE-cadherin allowed the detection of mosaic expression in endothelial cells.

Mosaicism was around 9% (Figure S2). Coinjection of the rescuing construct together with *VEcad*MO at low dose (MO2, 0.4 ng) resulted in reduction in the percentage of embryos that presented cranial hemorrhages or severe phenotype to 40% (n = 205) in comparison to 77% of the embryos injected with MO2 alone (n = 128) (Figure S2).

### The lack of blood flow partially explains the altered lumen formation in VE-cadherin morphants

**Figure 6:**
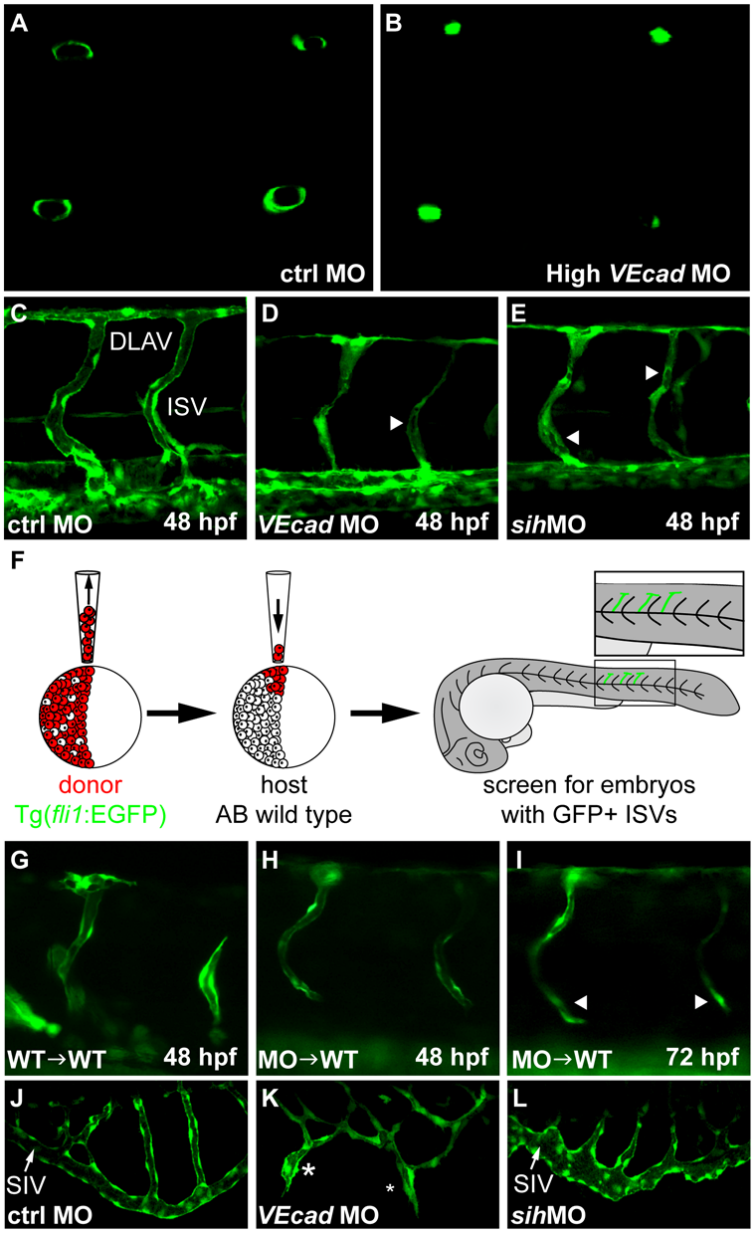
VE-cadherin expression influences lumen formation. (A–B) Optical cross sections of intersegmental vessels (ISVs) at 48 hpf in Tg(*fli1*:EGFP) background acquired at the confocal microscope by positioning the embryo on a dorsal orientation. CtrlMO injected embryos have fully lumenized ISVs (A) while *VE-cadherin* severe morphants (High *VEcad*MO) do not (B). (C–E) Lumenization of ISVs is influenced by blood circulation. Lateral views of trunk vessels of ctrlMO (C), high *VEcad*MO (D) and *sih*MO (E) injected embryos at 48 hpf. At this stage, ISVs and the dorsal longitudinal anastomotic vessels (DLAV) are fully lumenized in control embryos. In contrast, *VE-cadherin* and *sih* morphants present only a very small lumen in some portions of the ISVs (arrowheads in D and E) and essentially no lumen in DLAV. (F–I) Transplantation experiments. (F) Scheme of the experimental procedure. Donor cells from *fli1*:EGFP embryos were transplanted into AB wild type embryos at 4 hpf and, after development, embryos with EGFP positive ISVs were selected for analysis. When wild type embryos were used as donor (G), ISVs did form lumen and were integrated into the host vasculature at 48 hpf. Morphant donor cells transplanted into wild type embryos form thin lumens at 48 hpf (H) that fail to correctly integrate in the host vasculature and possibly collapse at 72 hpf (arrowheads in panel I). (J–L) Lumenization of subintestinal vein (SIV) is independent of blood flow as it occurs in the absence of heart beat in *sih* morphants at 72 hpf (L) but is dramatically affected by the absence of VE-cadherin. High *VEcad*MO SIVs display only partially lumenized regions which are not fully interconnected (asterisks in panel K).

Besides SV and CCV, other vessels showed profound alterations upon injection of high doses of *VEcad*MO. In addition to the defects observed in the cranial vasculature and the AAs, ISVs also presented a reduced or absent lumen at 48 hpf. Confocal analysis of dorsally oriented embryos allows the visualization of optical sections of the ISVs that express EGFP under the control of *fli1* promoter (Figure 6A,B). The embryos were studied at 48 hpf, since at this stage ISVs and DLAV are fully lumenized in control embryos, and confocal images illustrate the absence of lumen in most of the *VE-cadherin* morphant ISVs. An important question, however, is whether these alterations may be a direct effect of *VE-cadherin* knockdown or an indirect effect of the lack of circulation. To clarify this aspect we compared *VE-cadherin* morphants with *silentheart* (*sih*) morphants that lack blood flow due to complete impairment of cardiac contractility. We observed that, similarly to *VE-cadherin*, *sih* morphants show strongly reduced lumen in some portions of ISVs and fairly no lumens in DLAV at 48 hpf (Figure 6C–E). This suggests that the defect in lumen formation observed in *VE-cadherin* morphants might be, at least in part, explained by lack of flow. In transplantation experiments, donor cells from *fli1*:EGFP transgenic embryos were transplanted into wild type embryos at 4 hpf. At 24 hpf, embryos with GFP positive ISVs were selected for further analysis (Figure 6F). When *fli1*:EGFP wild type embryos were used as donors and AB wild type as recipients, ISVs did form lumen at 48 hpf and integrated in host vasculature (Figure 6G). When donor cells, derived from *fli1*:EGFP *VE-cadherin* morphants, were transplanted into wild type embryos they were able to form thin lumens at 48 hpf (Figure 6H) but they did not carry blood flow (direct observation). At 72 hpf the lumen of vessels formed by morphant cells collapsed (arrowheads in figure 6I) indicating that they failed to correctly connect to the host vasculature. Overall these data strongly suggest that VE-cadherin knockdown cells are able to incorporate into the wild type vasculature but are intrinsically defective and cannot maintain their adhesive interaction with the surrounding endothelial cells.

Furthermore, the analysis of other regions of the vascular tree such as the subintestinal vein (SIV) shows that lumen can be formed also in absence of blood flow as in *sih* morphants (Figure 6L) but these vessels, instead, are profoundly altered in *VE-cadherin* morphants. Only partially lumenized vessels are present (Figure 6K), but they do not fully connect to form the SIV.

### The vascular phenotype of VE-cadherin morphants is due to altered vascular connection and fusion

Considering the role of cadherins in cell differentiation [32] we asked whether *VE-cadherin* morphants show some defect in vascular gene expression. The analysis by whole-mount *in situ* hybridization shows that *VE-cadherin* morphants express endothelial markers without any detectable alteration (Figure S3). The normal expression of *dll4* and *ephB4a* suggests that arterial-venous differentiation is correct while expression of *gata1* and *scl1* in blood precursors at 20 hpf is consistent with the observation made in the mouse embryo where hematopoiesis was not affected [9], [10].

**Figure 7:**
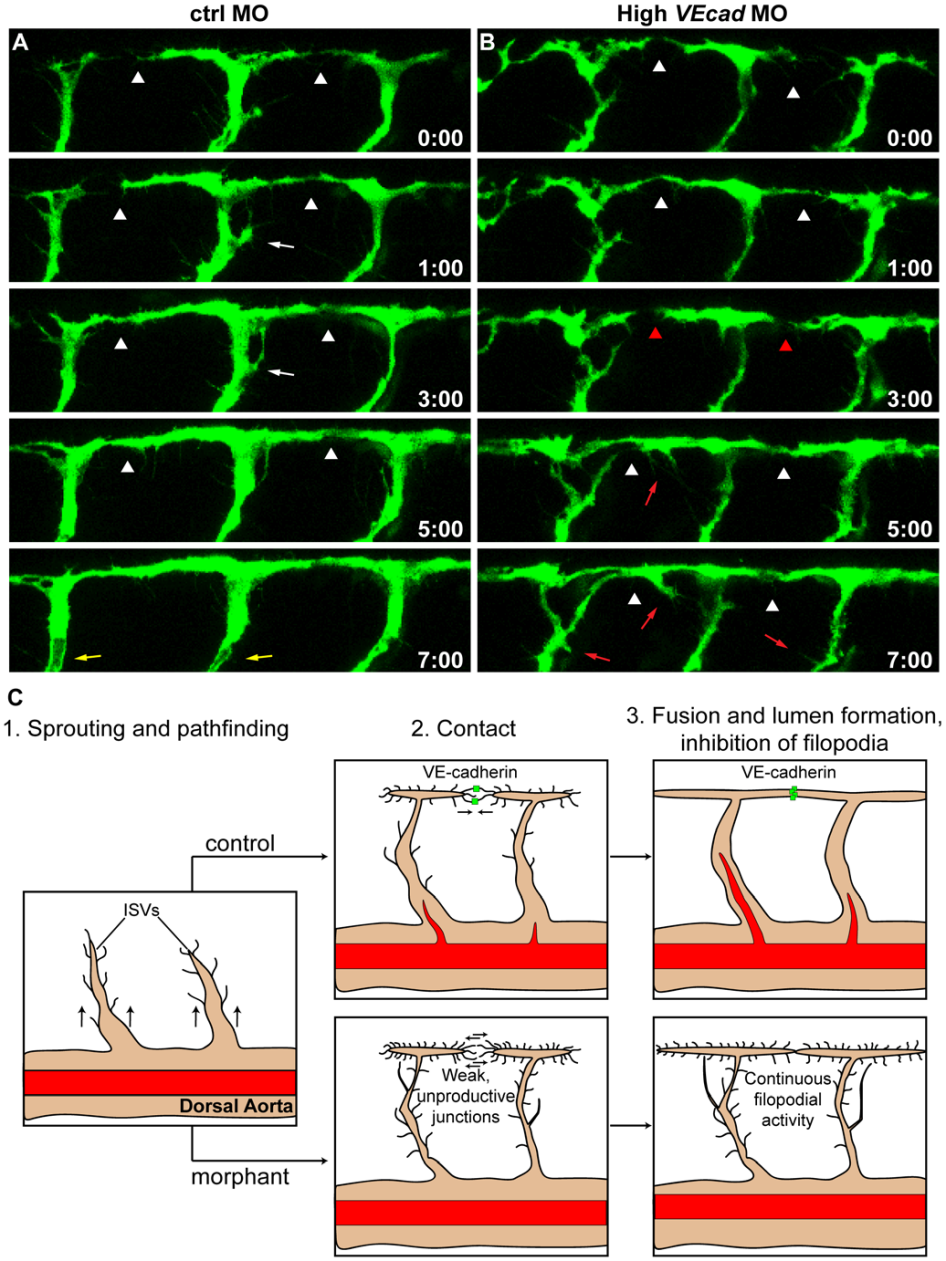
VE-cadherin is required for correct vascular connection and fusion. Time lapse analyses of ISVs of ctrl and high *VEcad*MO injected embryos from 28 to 36 hpf, performed in a *Tg*(*fli1*:EGFP) background. Lateral views of the trunk vessels are shown at the indicated time points (see Movies S7 and S8 for full time lapse). In ctrlMO injected embryos (A), dorsal extensions of ISVs that are about to form the DLAV (arrowheads) get stabilized soon after making contact with neighbouring vessels. In addition, the filopodial activity decreases once the connections are formed and stops before lumenization (white arrows). See lumen in ISVs at time point 7:00 (yellow arrows). In contrast, vessels of *VE-cadherin* severe morphants (B) show defects in establishing stable connections, as they make contact (arrowheads in B) but detach afterwards (red arrowheads). Moreover, the filopodial activity persists in ISVs even once they already appear dorsally interconnected (red arrows). (C) Model of the filopodial sprouting behaviour of control and *VE-cadherin* severe morphant ISVs.

Many of the vascular defects observed in *VE-cadherin* morphants may be explained by the lack of stable connections among endothelial cells in the different types of vessels. We therefore investigated this aspect more directly by the analysis of the dynamics of ISVs and DLAV formation *in vivo* by performing time-lapse analysis of their sprouting activity (Movies S7, S8 and figure 7). As previously reported [33], we observed that in control embryos, tip endothelial cells form many protruding filopodia which explore the sourrounding tissue. Once these protrusions touch other protrusions from a contiguous tip cell, they establish connections and the sprouting vessels subsequently fuse at the most dorsal region of the trunk to form DLAV. Interestingly, this process inhibits formation of new protrusions in the rest of the vessel. In contrast, embryos injected with high doses of *VEcad*MO show a significant delay in the establishment of anastomosis between contiguous ISVs, and the protruding filopodia touch and retract several times before establishing a connection. Most importantly, morphant ISVs maintain significant filopodia activity even after reciprocal connections are apparently established (Figure 7 and Movie S8). The sprouting activity is maintained until 46 hpf (not shown) while in controls it ceases within 36 hpf. These observations are consistent with the effect of *VEcad*MO observed in other types of vessels such as those of the head which present lack of correct connections and strong sprouting activity along their length (Figure 4I,J). No significant difference in the sprouting filopodia of ISVs was observed in *sih* morphants when compared to controls (M.M-B., unpublished observations).

## Discussion

Intercellular junctions in endothelial cells are important in the control of permeability to cells and plasma proteins but also for the correct architecture of the vascular network. The genetic inactivation in the mouse embryo of members of endothelial AJ such as VE-cadherin [9], [10], β-catenin [34], p120-catenin [35], VE-PTP [36], and others, results in early fetal lethality. In these mutants, after the initial patterning and formation of the primitive vascular plexus, the vessels do not further differentiate and tend to collapse and regress.

However, the lack of vascular remodeling appears to be a common feature of the inactivation of several genes expressed in the vascular system [3]. It is therefore difficult to identify, in the mouse embryo, the specific activity of VE-cadherin and the precise step of vascular development in which it is involved.

In this work, using zebrafish embryos, we studied the role of partial and complete VE-cadherin inactivation on development and maintenance of the vascular network. This model system is particularly suited to the study of VE-cadherin function, since the corresponding zebrafish orthologue maintains, as in the mouse, endothelial specific expression.

Using two different morpholinos we were able to define several novel and specific activities of VE-cadherin on vascular development. We found that VE-cadherin is not required for a correct patterning of the vasculature. Trunk and head vessels appear to be able to branch, and correctly follow elongation pathways. However, the absence of VE-cadherin inhibits the establishment of successful vessel contacts and fusion which are necessary steps for lumen formation. Several vessels lack connection with the rest of the vasculature and present reduced lumen and signs of initial regression. At high *VEcad*MO concentrations CCV cannot connect with the endocardium leaving the heart separated from the rest of the circulatory system.

The lack of blood flow contributes but does not fully explain the altered vascular lumenization in *VE-cadherin* morphants as suggested by comparison with *sih* phenotype. While ISVs lacked lumen in both types of morphants, SIV in *sih* morphants show the presence of a fully connected lumen which was instead strongly reduced or absent in *VE-cadherin* morphants. These data are consistent with the observations reported in *VE- cadherin* null mouse embryos [9] where, in large vessels, the vascular lumen was reduced or absent even before the establishment of flow.

Blood flow and shear stress are important factors for vascular remodeling [37]. Flow is important in the formation of stable vessels that integrate in a functional network [16], [33] and seems to be required in certain vessels (ISVs) for lumen opening. From the data presented here we propose that the role of VE-cadherin in lumen formation may be mediated, directly, by flow-independent induction of cell polarization and, indirectly, by promoting a correct connection between the sprouting vessels which is required for establishment of flow and final reshaping of the lumen.

During vascular development the first vessels which form in the embryo arise through vasculogenesis. This process involves endothelial cell differentiation, migration and assembly in tubular structures. After establishment of the axial blood vessels, smaller vessels such as the ISVs form through sprouting from the existing large vessels [16], [38]. This process has been studied in detail and includes endothelial cell migration towards the concentration gradient of growth factors such as vascular endothelial growth factor [39]. Migration is directed by “leader” endothelial cells that, similarly to branching morphogenesis in other tissues, direct the movement of the cells of the stalk. The leader endothelial cells form many filopodia at the leading edge which explore the sourrounding environment [38], [40]. When the leader cells encounter other sprouting vessels they fuse and form a communicating lumen. Upon fusion, the number of protruding filopodia rapidly decreases and the vessels form an interconnected vasculature (see figure 7C).

While the molecular pathways which regulate vascular sprouting and patterning have been described to a relatively large extent, very little is known about the molecules implicated in vascular fusion and lumen formation [38]. The present work shows for the first time that VE-cadherin plays an important role in these developmental steps. Most of the vascular defects observed in *VE-cadherin* morphants may be explained by the lack of strong intercellular contacts. When VE-cadherin is reduced or absent, contacts among vessels are weak and vessel fusion is delayed or absent (Movies S7, S8 and figure 7). In few regions such as the head vasculature or ISVs after the small vessels come in contact they maintain sprouting filopodia (figure 4J and 7B) strongly suggesting that the interconnection is incomplete and that the signals which follow vascular fusion are insufficient to abrogate sprouting. In other regions like the CCV and SV, the connection is unsuccessful and the vessels detach and eventually regress (Figures 5E–F).

In the isolated and cultured mouse allantois, in absence of VE-cadherin, the vessels form a primitive vascular network but they fail to organize stable connections, the vascular rudiments regress and the network collapses [41]. This occurs in the absence of flow and supports the concept that VE-cadherin plays an important role in the early organization of vascular like structures also in the absence of mechanical forces.

The abrogation of VE-cadherin in the mouse embryo leads to endothelial cell apoptosis in a report [9] but not in another [10]. In the present work we show that apoptosis was very limited in endothelial cells. A conceivable explanation is that apoptosis of endothelial cells is more apparent when the vascular phenotype is more severe as in Carmeliet et al [9]. In the fish the vascular pattern is rather well preserved and the observed phenotype is mild as compared to VE-cadherin gene inactivation in the mouse embryo. This likely explains the reduced apoptotic signal in vascular endothelium.

In summary, the present study introduces novel findings which clarify to a good extent the mechanism of action of VE-cadherin in promoting vascular development and which could not be observed in the mouse in earlier studies.

In general, VE-cadherin is a key protein in promoting early stages of vascular connection and fusion. This functional aspect of the protein fits with our knowledge on its mechanism of action in *in vitro* cell culture systems. Several published reports point to a role of VE-cadherin in transferring signals which promote junctional integrity and vascular stability [1]. VE-cadherin engagement inhibits endothelial cell growth, apoptosis and motility and upregulates a set of genes such as claudin-5 which contributes to tight junction organization [32]. We report here for the first time that VE-cadherin engagement may exert a negative feedback mechanism in reducing formation of sprouting filopodia. We still do not know the exact mechanism which regulates this inhibitory process but a recent report [42] shows that small GTPases, which are regulated by VE-cadherin, play a role through reshaping of the cell cytoskeleton.

Another novel observation resulting from the present work is that also a partial reduction in *VE-cadherin* expression may induce important defects in vascular integrity such as cranial hemorrhages and increase in vessel permeability. These defects were not observed in heterozygous mice where *VE-cadherin* expression was reduced by 50% [9], [10]. We cannot exclude, however, that stress conditions such as alterations in blood flow or the combined reduction of other related junctional proteins may cause hemorrhages also in heterozygous mice. It has recently been found in zebrafish that individual knockdown of genes implicated in junction stability such as *Rap1b*, *Pak2a*, *β-Pix* or *ccm1/krit* cause hemorrhagic phenotypes [43]–[45]. Strikingly, the combined knockdown of these genes synergizes and results in higher incidence of intracranial hemorrhages [45]. Importantly, all these genes are directly or indirectly associated to VE-cadherin and likely play a role in AJ stabilization. Thus, also partial alterations in expression or function of endothelial AJ proteins may result in high frequency of hemorrhagic events and altered control of permeability as we observed in the present study.

Finally, a new finding reported here is that the common cardinal vein lacks a correct connection with the endocardium leaving the heart separated from the rest of the circulatory system. As far as our knowledge it is the first time that this has been reported for VE-cadherin. The lack of closure of the circulatory loop may explain some downstream defects described in this phenotype such as the lack of a correct lumen formation.

## Supporting Information

Figure S1 Endothelial cell death is not increased in VE-cadherin endothelial cells. (A–D) TUNEL assay of 48 hpf embryos in a fli1:EGFP background allows the colocalization analysis of apoptotic cells (in red) and endothelial cells (in green). (A–B) Confocal images of the trunk region at the level of the urogenital opening are shown. VE-cadherin severe morphants (B) exhibit increased apoptosis in the blood island (arrowhead) where the blood cells and hematopoietic precursors (some of them fli1:EGFP positive) accumulate in the absence of circulation (arrowhead). In addition, increase in the number of apoptotic cells is observed in the tissues surrounding the ISVs and DLAV (arrows). Few vascular endothelial cells (asterisks) show increase in TUNEL staining. (C–D) Fluorescence microscopy images of the endothelial cells in the sinus venosus.
(0.08 MB PDF)

Figure S2 Endothelial specific expression of murine VE-cadherin rescues the phenotype of VE-cadherin morphants. A plasmidic DNA construct containing murine VE-cadherin cDNA under the zebrafish fli1 enhancer/promoter was coinjected with 0.4 ng of MO2 in order to rescue the hemorrhagic phenotype observed in the mild morphants. Three independent experiments were performed and the phenotype of morphant and rescued embryos was examined under the microscope at 54 hpf. The graph in panel A shows the mean percentage of embryos showing a morphant phenotype when injected with morpholino (VEcadMO) or morpholino plus plasmid (VEcadMO+mVEcad cDNA). The number of embryos analyzed is shown in table I. (B–D) Confocal images. Percentage of mosacism in 6 pairs of intersegmental vessels (ISV) located in the area of the trunk shown in figure B is displayed in table II. Average number of endothelial cells (EC) in 12 ISV+DLAV per embryo (at 48 hpf, n = 12, range 34 to 40) was counted in the Tg(fli1:nEGFP)y7 transgenic line, which shows EGFP-positive EC nuclei. Average number of EC expressing the transgenes was counted in AB embryos injected with a mixture of VEcadMO and cDNAs as indicated in the table. ECs expressing EGFP were counted in the same reference area at 48 hpf (n = 40, range 1 to 10). Percentage of mosaicism indicates the average proportion of EC cells expressing the transgene in 12 ISV+ DLAV. Figures C and D show mosaic expression of the EGFP transgene in ECs. Insets show co-expression of EGFP and murine VEcadherin:mCherry in the same ECs.
(0.15 MB PDF)

Figure S3 VE-cadherin knockdown does not affect vascular gene expression. In situ hybridization with pan-endothelial (A–B), arterial (C–D), venous markers (E–F) and hematopoietic precursor markers (G–J). Lateral views of the trunk region of 20 hpf embryos are shown, except A, B that report 24 hpf. Embryos injected with high dose (8 ng) of control or VE-cadherin morpholinos (high VEcadMO) were hybridized with the indicated probes (kdrb, dll4, ephB4a, gata1, scl1). High VEcadMO injected embryos (B, D, F, H, J) did not present differences in the expression of these markers when compared to control injected embryos (A, C, E, G).
(0.10 MB PDF)

Movie S1 Normal heart beat and blood circulation in control embryo at 30 hpf. Lateral view, anterior to the left. The heart beats regularly, and cranial and trunk circulation is correctly established
(1.83 MB MOV)

Movie S2 Trunk circulation in control embryo at 30 hpf. Lateral view, anterior to the left. Blood cells flow caudally in the dorsal aorta (upper vessel) and return back through the posterior cardinal vein (lower vessel).
(1.50 MB MOV)

Movie S3 Heart and blood circulation in mild morphant embryo at 30 hpf. Lateral view, anterior to the left. The heart beats regularly and the first circulatory loops in head and trunk are established. Notice some blood cells that tend to accumulate in the eye.
(1.62 MB MOV)

Movie S4 Trunk circulation in mild morphant embryo at 30 hpf. Lateral view, anterior to the left. Circulation is normally established but circulating blood cells are reduced in number.
(1.67 MB MOV)

Movie S5 Absence of circulation in severe morphant embryo at 30 hpf. Lateral view, anterior to the left. The heart beats regularly but the circulatory loops are not established. Blood circulation is absent in the head and trunk.
(1.88 MB MOV)

Movie S6 Absence of circulation in the trunk of severe morphant embryo at 30 hpf. Lateral view, anterior to the left. Blood cells accumulate in the vessels of the trunk and do not circulate.
(1.69 MB MOV)

Movie S7 ISVs sprouting in control embryos. Time-lapse movie. Lateral view of the trunk region, anterior to the left. Tg(fli1:EGFP) line was used. Movie starts at 28 hpf (0:00) and ends at 36 hpf (8:00). Dorsal extensions of ISVs send out filopodial protusions that upon contact to neighbouring ISVs remain connected. Filopodial activity decreases progresively and ceases just before lumen formation.
(4.82 MB MOV)

Movie S8 ISVs sprouting in morphant embryos. Time-lapse movie. Lateral view, anterior to the left. Tg(fli1:EGFP) line was used. Movie starts at 28 hpf (0:00) and ends at 36 hpf (8:00). ISVs present a large number of branches and filopodial protusions that make contact with a neighbouring sprouting vessel but detach and retract afterwards. Endothelial cells in morphant ISVs show a highly motile behaviour and the sprouting filopodial activity persists after the vessels are apparently connected.
(4.09 MB MOV)
