# Supplementary figures and images for: Stable Vascular Connections and Remodeling Require Full Expression of VE-Cadherin in Zebrafish Embryos
Source: PLoS One. 2009 Jun 3;4(6):e5772. doi: 10.1371/journal.pone.0005772 (PMC2685470; doi:10.1371/journal.pone.0005772)

Figure S1. Montero-Balaguer et al.

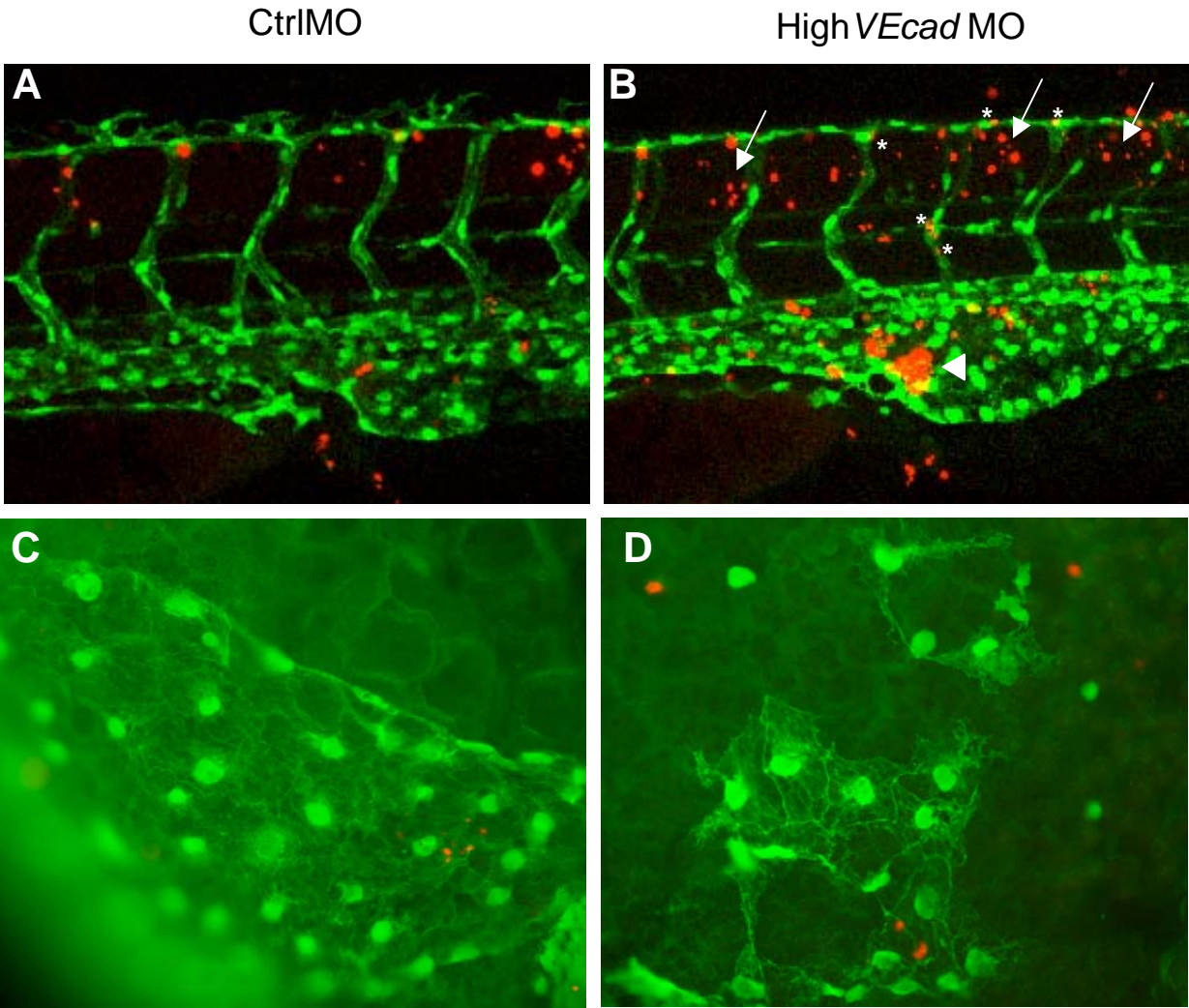

Supplement: Figure S1 — Endothelial cell death is not increased in VE-cadherin endothelial cells. (A–D) TUNEL assay of 48 hpf embryos in a fli1:EGFP background allows the colocalization analysis of apoptotic cells (in red) and endothelial cells (in green). (A–B) Confocal images of the trunk region at the level of the urogenital opening are shown. VE-cadherin severe morphants (B) exhibit increased apoptosis in the blood island (arrowhead) where the blood cells and hematopoietic precursors (some of them fli1:EGFP positive) accumulate in the absence of circulation (arrowhead). In addition, increase in the number of apoptotic cells is observed in the tissues surrounding the ISVs and DLAV (arrows). Few vascular endothelial cells (asterisks) show increase in TUNEL staining. (C–D) Fluorescence microscopy images of the endothelial cells in the sinus venosus. (0.08 MB PDF) [file pone.0005772.s001.pdf]

Figure S3. Montero-Balaguer et al.

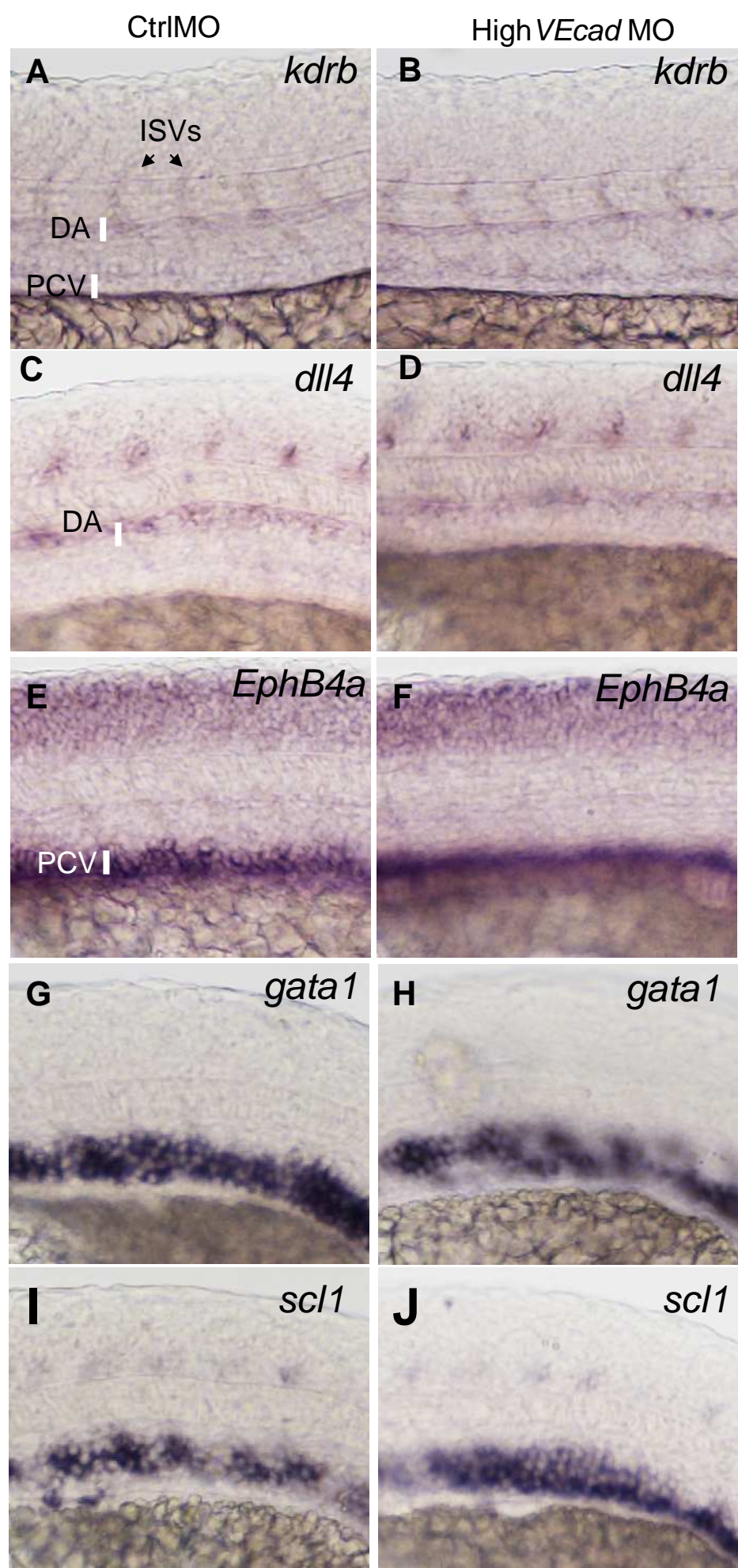

Supplement: Figure S3 — VE-cadherin knockdown does not affect vascular gene expression. In situ hybridization with pan-endothelial (A–B), arterial (C–D), venous markers (E–F) and hematopoietic precursor markers (G–J). Lateral views of the trunk region of 20 hpf embryos are shown, except A, B that report 24 hpf. Embryos injected with high dose (8 ng) of control or VE-cadherin morpholinos (high VEcadMO) were hybridized with the indicated probes (kdrb, dll4, ephB4a, gata1, scl1). High VEcadMO injected embryos (B, D, F, H, J) did not present differences in the expression of these markers when compared to control injected embryos (A, C, E, G). (0.10 MB PDF) [file pone.0005772.s003.pdf]
