# Supplementary material for: Stable Vascular Connections and Remodeling Require Full Expression of VE-Cadherin in Zebrafish Embryos
Source: PLoS One. 2009 Jun 3;4(6):e5772. doi: 10.1371/journal.pone.0005772 (PMC2685470; doi:10.1371/journal.pone.0005772)
Supplement: Figure S2 — Endothelial specific expression of murine VE-cadherin rescues the phenotype of VE-cadherin morphants. A plasmidic DNA construct containing murine VE-cadherin cDNA under the zebrafish fli1 enhancer/promoter was coinjected with 0.4 ng of MO2 in order to rescue the hemorrhagic phenotype observed in the mild morphants. Three independent experiments were performed and the phenotype of morphant and rescued embryos was examined under the microscope at 54 hpf. The graph in panel A shows the mean percentage of embryos showing a morphant phenotype when injected with morpholino (VEcadMO) or morpholino plus plasmid (VEcadMO+mVEcad cDNA). The number of embryos analyzed is shown in table I. (B–D) Confocal images. Percentage of mosacism in 6 pairs of intersegmental vessels (ISV) located in the area of the trunk shown in figure B is displayed in table II. Average number of endothelial cells (EC) in 12 ISV+DLAV per embryo (at 48 hpf, n = 12, range 34 to 40) was counted in the Tg(fli1:nEGFP)y7 transgenic line, which shows EGFP-positive EC nuclei. Average number of EC expressing the transgenes was counted in AB embryos injected with a mixture of VEcadMO and cDNAs as indicated in the table. ECs expressing EGFP were counted in the same reference area at 48 hpf (n = 40, range 1 to 10). Percentage of mosaicism indicates the average proportion of EC cells expressing the transgene in 12 ISV+ DLAV. Figures C and D show mosaic expression of the EGFP transgene in ECs. Insets show co-expression of EGFP and murine VEcadherin:mCherry in the same ECs. (0.15 MB PDF) [file pone.0005772.s002.pdf]

Figure S2. Montero-Balaguer et al.

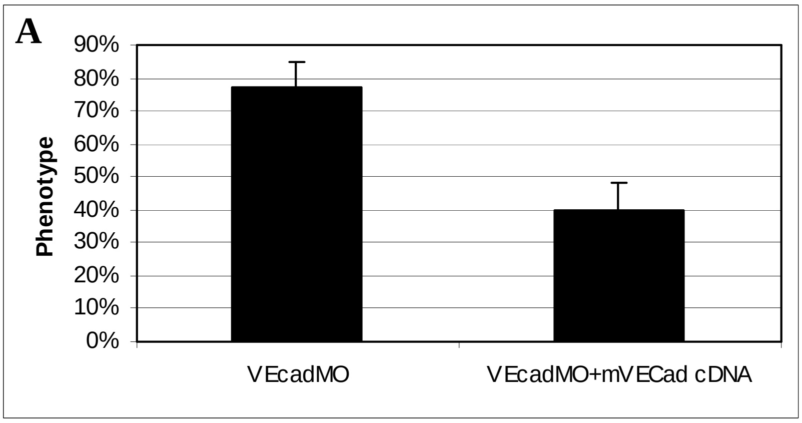

Table I-Rescue injections

Injected material	% of embryos with phenotype	No. of embryos with phenotype	No. of injected embryos
*VEcad*MO	77	100	128
*VEcad*MO+m*VEcad* cDNA	40	78	205

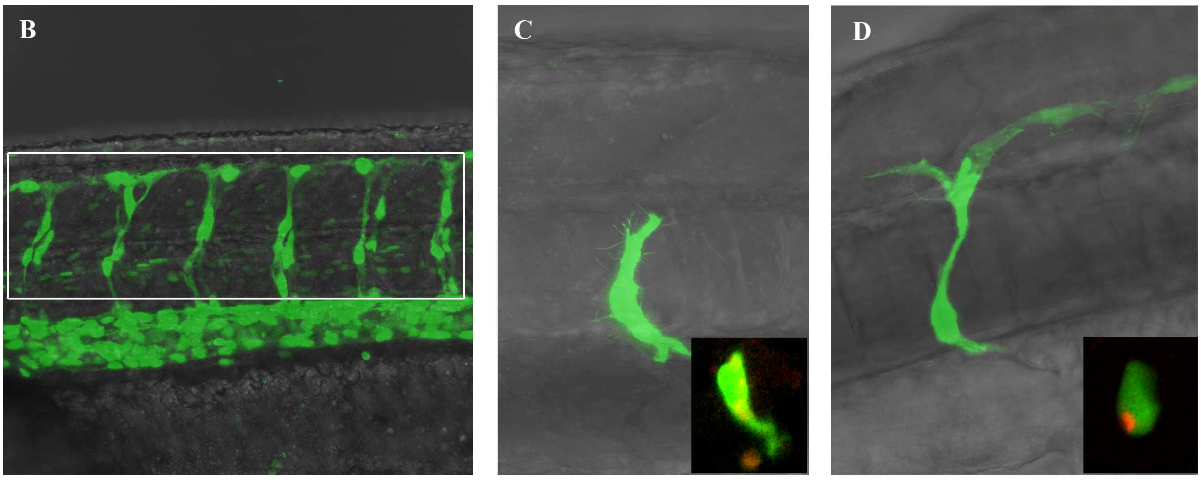

Table II- Percentage of mosaicism

Injected material	Average No. EC per reference area	Average No. EC with transgenes	% mosaicism
*VEcad*MO+m*VEcad* cDNA+GFP	37	3.5	9.45
